# Smoking Relapse Causing an Acute Exacerbation of Desquamative Interstitial Pneumonia with Pleural Effusions and Mediastinal Adenopathies

**DOI:** 10.1155/2018/8503694

**Published:** 2018-06-26

**Authors:** Tyler Pickell, Jamie Donnelly, Francois Abi Fadel

**Affiliations:** ^1^Marian University College of Osteopathic Medicine, Indianapolis, IN, USA; ^2^Ameripath, Indianapolis, IN, USA; ^3^Respiratory Institute, Cleveland Clinic, Cleveland, OH, USA

## Abstract

Desquamative interstitial pneumonia (DIP) is a rare interstitial pneumonia often caused by smoking. DIP is typically regarded as a chronic disease, but acute DIP exacerbations can occur, and some have resulted in death. Factors that can provoke a DIP exacerbation are not well described in the literature. We present a case of a 58-year-old male with DIP, who after being treated successfully with smoking cessation and steroids for 7 months, required hospitalization for acute hypoxemic respiratory failure. This acute episode was very likely an exacerbation of his DIP after a smoking relapse period of 6 weeks prior to this acute presentation. This report also highlights unique CT findings in a DIP case of pleural effusions and mediastinal adenopathies seen chronically and relapsing acutely. To the best of our knowledge, CT findings of pleural effusions and mediastinal adenopathies concurrently have not been described in a case of DIP in chronic or acute conditions.

## 1. Introduction

Desquamative interstitial pneumonia (DIP) is one of the rarest interstitial pneumonias originally described by Liebow in 1965 [1, 2]. Liewbow et al.'s paper first described DIP cases with acute exacerbations that resulted in death. Currently, literature is lacking on potential causes leading to DIP exacerbations, and very few cases of DIP exacerbations have been described [3–7].

We are reporting a case of a 58-year-old male with proven DIP on prior surgical lung biopsy, who presented with acute fulminant respiratory failure. Unique features of this case are first that the respiratory failure resulted most likely from a DIP exacerbation, which was very likely due to smoking relapse. In addition, our patient had significant pleural effusions and mediastinal adenopathies that were not explained by any other etiologies and we believe were due to an acute decline of DIP.

## 2. Case Report

**Figure 1:**
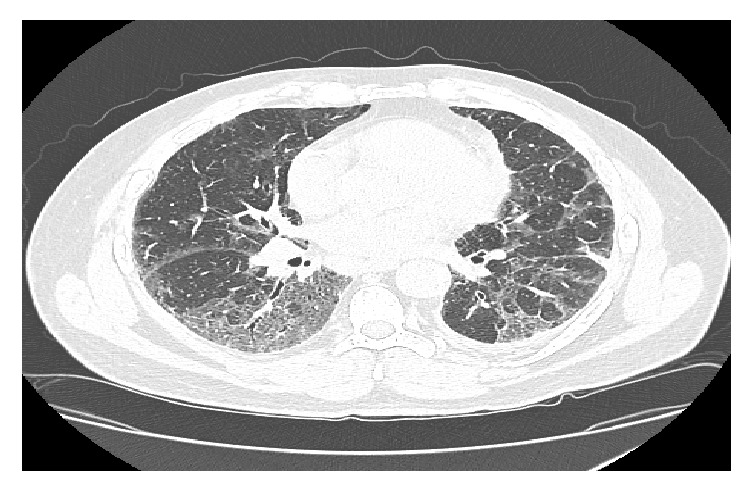
CT-high resolution chest without contrast. Taken 15 months before acute exacerbation. Ground glass opacities with thickening of the interlobular septa, predominantly in the lung bases.

Our patient is a 58-year-old Caucasian male referred initially to the pulmonary clinic for an abnormal high resolution computed tomography (HRCT) of the chest (Figure 1) showing ground glass opacities (GGOs), thickening of the interlobular septa primarily in the bases with minimal honeycombing, mediastinal adenopathies, and small bilateral pleural effusions. He had complaints of progressive shortness of breath and an unproductive cough. Medical history was relevant for 31-pack-year smoking history, asbestos exposure, uncontrolled Diabetes Mellitus type II, and obesity. His oxygen saturation was 93% on room air. Lung auscultation revealed bibasilar crackles with poor air entry but no clubbing or cyanosis. Pulmonary function test demonstrated an obstructive lung disease with mildly decreased forced vital capacity (FVC) at 76% and forced expiratory volume in one second (FEV1) at 70%, borderline FEV1/FVC ratio at 72%, and excellent effort. Lung volumes also revealed a restrictive lung disease with moderately decreased total lung capacity (TLC) at 64%. Finally, the diffusion capacity of carbon monoxide (DLco) was severely reduced at 44%.

The initial outpatient workup included a complete blood count (CBC), comprehensive metabolic panel (CMP), erythrocyte sedimentation rate (ESR), N-terminal probrain natriuretic peptide (NT-proBNP), urinalysis, lactate dehydrogenase (LDH), C-reactive protein (CRP), erythrocyte sedimentation rate (ESR), creatine kinase (CK), angiotensin converting enzyme (ACE) level, antineutrophil cytoplasmic antibodies (ANCA), rheumatoid factor (RF), anti-cyclic citrullinated peptide immunoglobulin G (Anti-CCP), histoplasmosis antibodies, human leukocyte antigen B27 (HLA-B27), aspergillus galactomannan antigen, interferon-gamma release assay for tuberculosis, anti-SCL-70 antibody, beta D-glucan, and antinuclear antibodies (ANA). All were negative except a borderline nonspecific elevation in ACE, LDH, and CK levels and positive ANCA antibodies.

**Figure 2:**
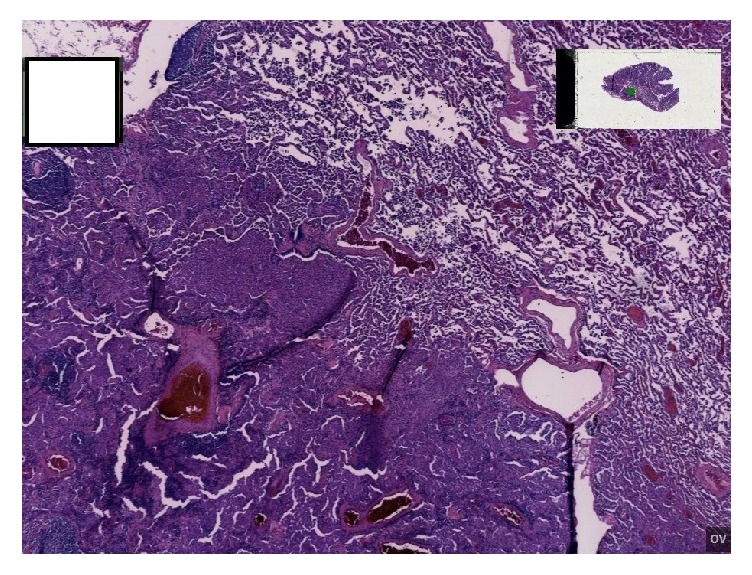
Low power overview showing DIP and RB-ILD with adjacent normal but slightly inflamed lung.

**Figure 3:**
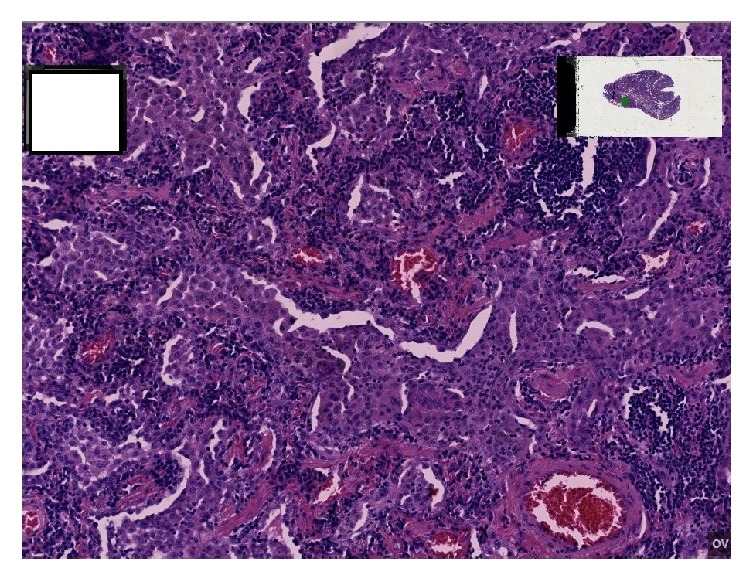
10x lung view showing dense accumulation of intra-alveolar macrophages. Classic histological DIP findings.

To address the interstitial lung disease pattern, a video-assisted thoracoscopic surgery (VATS) lung biopsy was performed. Right lower and right upper lobe wedge excisions revealed classic desquamative interstitial pneumonia, along with foci of respiratory bronchiolitis interstitial pneumonia, mild interstitial fibrosis, and emphysematous changes (Figures 2 and 3).

The patient's history of lifelong smoking along with asbestos exposure [8] likely contributed to these histological changes and DIP diagnosis.

To treat DIP, he was started on a tapered dose of glucocorticoids (starting at 40 mg of prednisone) along with nicotine replacement therapy for smoking cessation. Over a 7-month treatment course, the prednisone dose was lowered to 10mg with improvement in his dyspnea and cough. With assistance from the nicotine replacement, the patient successfully quit smoking for 6 months. Repeat CT images showed improvement in GGOs and resolution of prior small pleural effusions.

After 7 months of DIP treatment and with minimal respiratory symptoms, he restarted smoking one pack of cigarettes a day. Six weeks after restarting smoking, respiratory issues quickly developed, and he required hospitalization for acute hypoxemic respiratory failure. Upon admission the patient was tachypneic with labored breathing, worsening bibasilar crackles, decreased air entry, and an unproductive cough. Given his presentation and dropping oxygen saturation to 84% on room air, he was given 100% FiO2 (fraction of inspired oxygen) by nonrebreather mask.

After discussing treatment options for respiratory failure, the patient refused intubation and mechanical ventilation with complete understanding that his condition could be life-threatening. His breathing was therefore supported with alternating 100% FiO2 by nonrebreather mask and noninvasive ventilator support to maintain oxygen saturation above 90%.

**Figure 4:**
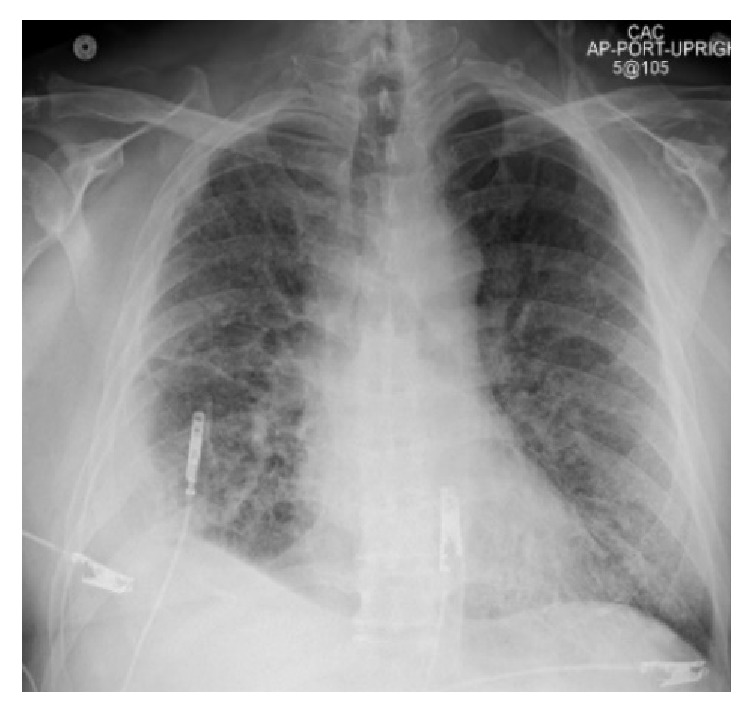
Anteroposterior chest X-ray taken at the hospital admission during acute exacerbation of DIP. Right pleural effusion and bilateral lung interstitial opacities.

**Figure 5:**
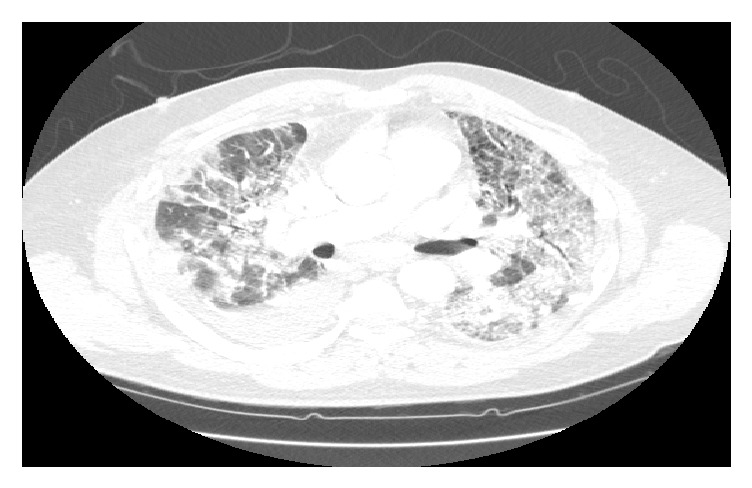
Chest CT angiogram taken during the acute exacerbation. Right pleural fluid filling 1/4 of chest, bilateral interstitial, and airspace disease. Ground glass opacities, pathological adenopathies in the mediastinum, cardiomegaly, and no pulmonary embolus.

A chest X-ray (Figure 4) and a chest CT angiogram (Figure 5) were ordered. No pulmonary emboli were found. However, imaging did show significant worsening bilateral GGOs, bibasilar consolidations, mediastinal adenopathies, and a new moderate right pleural effusion. The patient was started on broad spectrum antibiotics with vancomycin, piperacillin/tazobactam, and azithromycin. Laboratory workup was unremarkable for infection however. White blood cell count was slightly elevated at 12,600 without a left shift. Procalcitonin level was borderline at 0.14 ng/mL, C-reactive protein was elevated initially but then normalized, and the patient was afebrile at 98.4 Fahrenheit. NT-proBNP was normal which excluded congestive heart failure as a cause for the pleural effusion and GGOs. Repeated arterial blood gas measurements displayed no abnormalities in pH or carbon dioxide levels despite his labored breathing.

Additional negative tests included respiratory polymerase chain reaction viral panel, fungal serology, blood cultures, sputum acid-fast bacilli testing, urine streptococcus, and legionella. Sputum fungal and bacterial cultures grew* Candida dubliniensis* and* Pseudomonas fluorescens*. Although, these organisms are not generally considered pathogens in humans unless severely immunocompromised and have been found in normal oral flora [9, 10].

To further investigate a cause for the acute decompensation, the patient was offered a bronchoscopy and thoracentesis for mediastinal sampling and pleural effusion, respectively. After an extensive discussion, he refused all invasive diagnostic procedures.

Four days passed after being given broad spectrum antibiotics and no improvement was observed. Repeat imaging showed a persistence of bilateral interstitial opacities and pleural effusion. His oxygen saturation level still required 100% FiO2 supplementation to maintain above 90%. Because initial workup for the acute respiratory failure was inconclusive, less common etiologies were considered including DIP exacerbation.

On the fourth hospitalization day, treatment for possible DIP exacerbation was initiated and antibiotics were stopped. He was given 40 mg methylprednisolone every 8 hours. Within hours, his shortness of breath and oxygen saturation drastically improved. All respiratory symptoms dissipated, and he was stable to be treated as an outpatient after 48 hours of starting treatment. His discharge plan included oxygen support by nasal cannula and oral prednisone. He agreed to a smoking cessation plan with nicotine patches.

**Figure 6:**
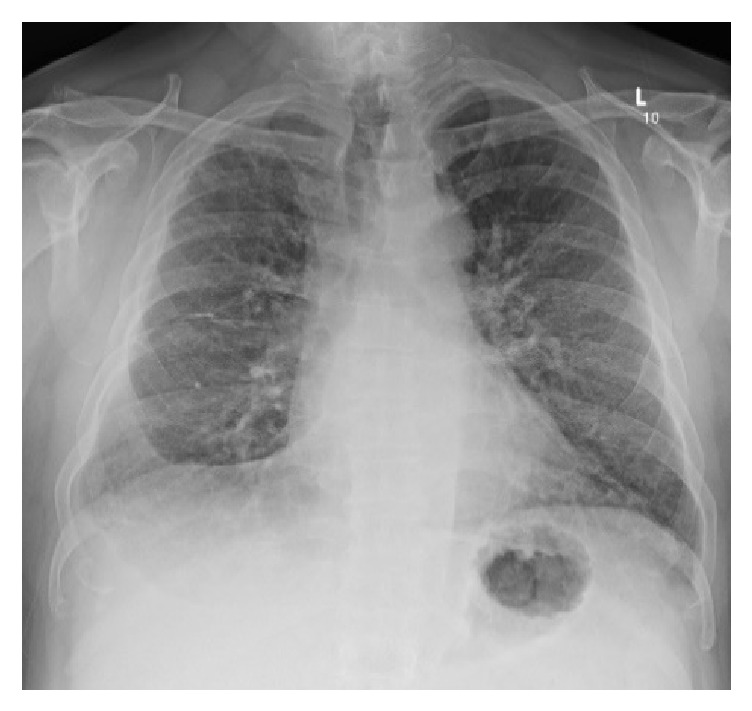
Anteroposterior chest X-ray 10 days after acute exacerbation and treatment with glucocorticoids. Right pleural effusion resolved with improvement in interstitial opacities.

Ten days after discharge, an office-visit chest X-ray (Figure 6) showed complete resolution of the pleural effusion and bilateral interstitial and airspace diseases markedly improved. His oxygen saturation climbed to 96% on room air indicating supplemental oxygen was no longer needed.

## 3. Discussion

DIP and respiratory bronchiolitis interstitial lung disease (RB-ILD) are the two interstitial pneumonias associated with smoking [1]. While 90% of DIP is primarily due to smoking, 10% is a result of other causes [1, 11–13]. Other causes include drugs (i.e., sirolimus and nitrofurantoin) [12], infections (i.e., cytomegalovirus, hepatitis C, and aspergillus) [12], connective tissue diseases, environmental exposures (i.e., asbestos, beryllium, and copper) [1, 11, 12], and in children with a surfactant protein mutation [12, 14]. Some documented causes of DIP exacerbations include lung biopsy with VATS and lung transplantation [5, 15, 16].

Common symptoms of DIP are dyspnea on exertion and a dry cough [1, 11]. Physical exam findings in DIP typically consist of bibasilar crackles and sometimes cyanosis or digital clubbing [11]. The typical age of onset is 40-60 [1, 11].

Despite similarities in presentation with other interstitial pneumonias, especially RB-ILD, histological as well as clinical correlation can be utilized to differentiate DIP [11, 13, 17]. The gold standard for DIP and RB-ILD diagnosis is surgical lung biopsy though both may exist together [1]. Histological features unique to DIP are as follows: accumulation of brown-yellow pigmented “smokers pigment” macrophages diffusely within alveolar spaces including distal airways, with associated interstitial inflammation and/or fibrosis, however without or with very few honeycomb changes [11, 12, 18, 19]. CT findings in DIP include patchy GGOs that are usually symmetric and in the mid and lower zones [11–13, 18]. PFTs demonstrate a restrictive lung defect with a decreased DLco [1, 12].

Though this patient was a heavy lifelong smoker, 6 months of smoking cessation followed by 6 weeks of smoking relapse was enough to significantly flare his DIP into a life-threatening event. To our knowledge, this is the first case to describe smoking relapse as a likely cause for DIP exacerbation. Research shows 54-67% of smokers who attempt to quit in the first-year relapse [20]. Given relapse is so prevalent among smokers attempting to quit, relapse smoking as a possible cause for DIP exacerbation is significant.

Additionally, there are only one prior case reporting mediastinal adenopathies [21] and no cases reporting pleural effusions with DIP as observed in our patient.

The two main treatments for DIP are smoking cessation and glucocorticoids. The DIP mortality rate is 30% at 10 years [17]. Appropriate treatment for an acute DIP episode may lead to complete recovery to baseline as illustrated in our case. Avoidance of possible causes for DIP exacerbations, such as relapse smoking, may lead to improved mortality outcomes in this patient population.

## 4. Conclusion

Desquamative Interstitial Pneumonia (DIP) can present with fulminant near fatal exacerbations. We believe that smoking relapse has contributed to the acute exacerbation of our patient's DIP. This underlines the importance of smoking cessation and preventing smoking relapse in all DIP patients as one of the few treatments that could alter the course and prevent exacerbations. Glucocorticoids remain the other treatment for chronic and fulminant cases of DIP. Although not previously described, our case demonstrates that pleural effusions and mediastinal adenopathies may exist in both acute and chronic DIP presentations.
